# Data-Driven Detection of Stealthy IA Attacks in Industrial Cyber-Physical Systems via DGM Quantification †

**DOI:** 10.3390/s26185750

**Published:** 2026-09-10

**Authors:** Jingzhao Chen, Bin Liu, Zhiqun Jiang

**Affiliations:** 1 Henan Engineering Research Center of Intelligent Manufacturing and Digital Twin, SIAS University, Zhengzhou 451150, China; jzh.chen@sias.edu.cn (J.C.); zhiqun.jiang@foxmail.com (Z.J.); 2 Engineering Research Center of Metallurgical Automation and Measurement Technology, Ministry of Education, Wuhan University of Science and Technology, Wuhan 430081, China

**Keywords:** industrial cyber-physical systems, data-driven detection, anomaly detection, subspace methods, stealthy attacks, false data injection

## Abstract

Industrial cyber-physical systems face increasing security threats from sophisticated cyber attacks. Traditional anomaly detectors generally require exact system models and noise statistics, which are often unavailable in practical industrial environments. To address this, this paper proposes a data-driven security detection framework based on the quantification of differences in generalized models (DGM). Utilizing only accessible operational data from the control layer, the method employs closed-loop subspace orthogonal projections to construct two detection variables: a static innovation sequence estimator and an extended dynamic Markov matrix estimator. The static estimator identifies fundamental model mismatches caused by standard denial-of-service and essential false data injection attacks. Meanwhile, the dynamic estimator successfully captures the structural distortions induced by advanced stealthy attacks that typically deceive Kullback–Leibler divergence detectors. The proposed methods were validated using a hardware-in-the-loop platform featuring a two degree-of-freedom robot and a DC servo motor. Experimental results confirm that the DGM framework effectively detects multiple types of stealthy integrity and availability (IA) attacks without relying on system parameters or degrading optimal control performance.

## 1. Introduction

Industrial cyber-physical systems (ICPSs) represent a class of intelligent systems characterized by the deep integration and real-time interaction between computing units and physical entities across diverse network environments [1,2,3]. By fundamentally transforming traditional manufacturing architectures, ICPSs have been widely deployed in critical national infrastructures, including smart grids, energy distribution, and advanced manufacturing [4,5]. However, this high degree of integration inherently expands the attack surface, allowing cyber threats to propagate directly from the network layer to the physical operational layer, thereby causing severe safety hazards. Recent real-world incidents, ranging from the Triton malware attack on safety instrumented systems [6] and the notorious Stuxnet manipulations [7] to the Florida water treatment plant breach [8] and sophisticated cross-regional attacks via Starlink satellite links [9], underscore the urgency and criticality of robust security defense mechanisms for ICPS.

To counteract these escalating threats, security defense strategies are generally classified into three paradigms: security prevention, resilient control, and detection–isolation mechanisms [10,11,12]. While prevention and resilient control are effective, they often fail when adversaries successfully bypass network firewalls or launch destructive attacks targeting the actuator channels. Consequently, anomaly detection acting as the core of the detection–isolation paradigm has emerged as the last and most vital line of defense [13]. Traditional detection methods, such as the widely applied $\chi^{2}$ and Kullback–Leibler divergence (KLD) detectors [14], typically rely on state estimators to analyze the statistical anomalies of the residual sequences [15,16,17]. However, these model-based methodologies suffer from a fundamental limitation: they mandate the exact knowledge of the physical plant’s mathematical model matrices and the precise statistical properties of process and measurement noises.

In practical industrial applications, especially those employing model-free, fuzzy, or non-linear control algorithms [18,19] alongside advanced privacy-preserving and unknown-input filtering schemes for complex physical environments [20], fulfilling such stringent modeling assumptions is highly unrealistic.

Furthermore, attackers continually evolve their strategies to evade detection, developing sophisticated stealthy attacks capable of bypassing residual-based monitors. To defend against advanced stealthy manipulations, such as replay and zero-dynamics attacks [21], researchers have proposed active defense techniques like physical watermarking [22,23] and random coding. Unfortunately, injecting exogenous noise signals or modifying hardware architectures inherently compromises the optimal control performance of the physical plant. Moreover, the deployment of watermarking techniques often creates coupling conflicts when multiple detectors are required to operate simultaneously.

To address the limitations of active defense, understanding and modeling the attack behaviors becomes paramount. In our previous work [24], we systematically investigated a multiplicative integrity and availability (IA) attack model. Unlike conventional additive false data injection (FDI) attacks that require continuous online signal generation, the multiplicative attack framework parameterizes the malicious actions as static deviations in the generalized system matrices. This model significantly reduces the adversary’s hardware costs and exposure risks, while satisfying the essential stealthiness constraint (i.e., avoiding obvious anomalies in the control layer monitoring). The emergence of such advanced attack frameworks necessitates the development of novel detection schemes that are independent of nominal system models and do not degrade the inherent control performance.

Driven by these challenges, data-driven security detection techniques leveraging historical operational data have gained significant attention. Our preliminary explorations [18,25] have demonstrated the feasibility of detecting attacks by evaluating the modal mismatches using subspace methods. Building upon the foundational conceptual framework proposed in our preliminary conference paper [26], this article systematically investigates a completely data-driven detection methodology and establishes a mature security defense framework. The primary similarity between this study and our previous explorations lies in the shared mathematical basis of utilizing historical operational data for subspace evaluations. However, the current manuscript introduces substantial theoretical advancements and empirical differences. The unique academic contributions of this study compared to previous works are summarized as follows:

1. We propose a unified data-driven anomaly detection framework that strictly utilizes accessible operational data sequences, effectively eliminating the restrictive dependency on exact physical plant parameters and noise statistical knowledge. Furthermore, the proposed DGM detectors do not require the injection of any exogenous signals, thereby ensuring zero degradation of the ICPS control performance.
2. Advancing beyond the basic static evaluations in our preliminary work, we derive an extended dynamic Markov matrix estimator and introduce a novel geometric mechanism analysis. This formal analysis elucidates how the dynamic estimator uniquely captures the rotational and stretching distortions induced by sophisticated FDI-II attacks that successfully deceive traditional KLD detectors.
3. We elevate the validation methodology from previous pure numerical simulations to comprehensive hardware-in-the-loop experiments. By developing a physical platform featuring a two degree-of-freedom robot and a DC servo motor, empirical results systematically validate the practicability and superiority of the proposed framework against denial-of-service attacks, essential FDI attacks, and highly stealthy FDI scenarios.

In summary, the primary theoretical contribution of this research lies in establishing a direct mathematical relationship between physical attack behaviors and the structural variations in the data subspace. Rather than simply providing an alternative numerical algorithm, the proposed framework explicitly quantifies how stealthy attacks alter the inherent system dynamics using strictly operational data. This provides a rigorous analytical foundation for designing security detectors in industrial cyber-physical systems where exact mathematical models are unavailable.

The remainder of this article is organized as follows: Section 2 formulates the system model and the multiplicative IA attacks. Section 3 details the data-driven DGM detection methodologies, including the derivations of the static and dynamic detection variables along with their geometric interpretations. Section 4 presents the experimental setup and analyzes the evaluation results. Finally, Section 5 concludes this paper.

## 2. Problem Formulation

**Figure 1:**
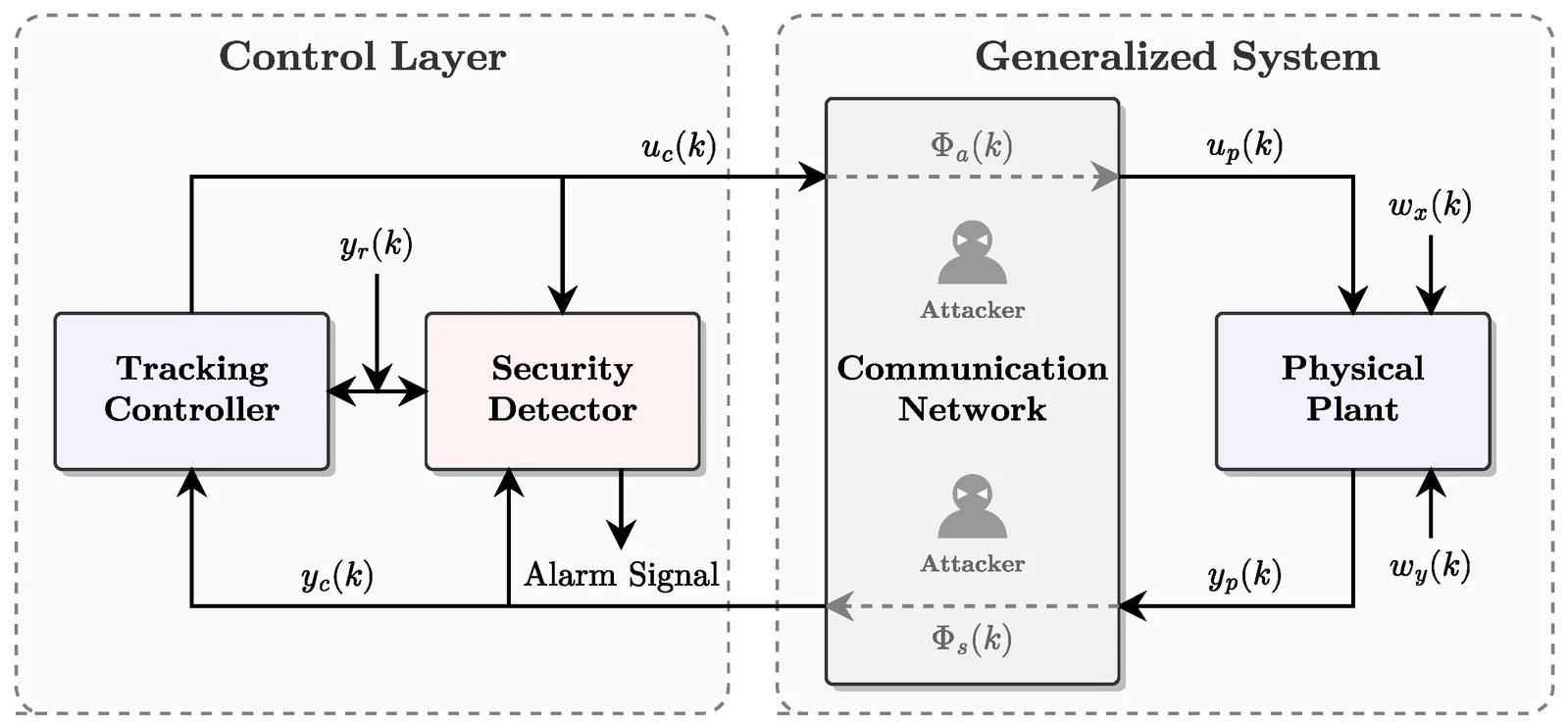
The tracking control ICPS deployed with a security detector.

As illustrated in Figure 1, the architecture of the considered ICPS primarily consists of three interacting components. These components are the control layer, the communication network, and the physical plant. From the defender’s perspective at the control layer, the physical plant and the communication network including potential attackers are collectively treated as a generalized controlled system. Specifically, $y_{r}\left(k\right)$ is the reference trajectory, $y_{c}\left(k\right)$ and $u_{c}\left(k\right)$ denote the received output and control input at the control layer, respectively. $\Phi_{a}\left(k\right)$ and $\Phi_{s}\left(k\right)$ represent the attacks injected into the communication network, while $u_{p}\left(k\right)$ and $y_{p}\left(k\right)$ are the actual input and output of the physical plant subjected to process noise $w_{x}\left(k\right)$ and measurement noise $w_{y}\left(k\right)$. To facilitate readability, the main mathematical symbols used in this section are summarized in Table 1.

### 2.1. System Description

The underlying physical plant is located within the physical layer, and its dynamics can be described by a discrete-time linear time-invariant stochastic state-space model given by

(1) $$\left\{\begin{array}{c} x_{p}\left(k+1\right)=A_{p}x_{p}\left(k\right)+B_{p}u_{p}\left(k\right)+w_{x}\left(k\right),\\ y_{p}\left(k\right)=C_{p}x_{p}\left(k\right)+w_{y}\left(k\right), \end{array}\right.$$

where $x_{p}\left(k\right)\in R^{n_{x}}$, $u_{p}\left(k\right)\in R^{n_{u}}$, and $y_{p}\left(k\right)\in R^{n_{y}}$ represent the system state, control input, and measurement output, respectively. The matrices $A_{p}$, $B_{p}$, and $C_{p}$ denote the model parameters, while $w_{x}\left(k\right)$ and $w_{y}\left(k\right)$ represent the noise signals.

For the controlled physical plant defined in Equation (1), we consider the following two assumptions that distinguish our work from the existing literature in the security detection field.

**Assumption** **1.** *The exact values of the system parameters $A_{p}$, $B_{p}$, and $C_{p}$ cannot be precisely known or are completely unknown to the defender. However, the dimensions of the system variables, namely $n_{x}$, $n_{u}$, and $n_{y}$, are known.*

**Assumption** **2.** *The process noise $w_{x}\left(k\right)$ and measurement noise $w_{y}\left(k\right)$ in Equation* (1) *are independent Gaussian white noises, but their corresponding statistical parameters are unknown.*

**Remark** **1.** *Systems satisfying the aforementioned two assumptions are common in practice, such as industrial control systems that utilize non-model-based controllers. Existing security detection methods are generally inapplicable under these two assumptions because they typically require exact knowledge of both the model parameters and the statistical properties of the noise. This reality motivates us to investigate a more practical data-driven security detection method. In this approach, the detector is constructed and deployed directly using the system’s operational data stored at the control layer, without relying on the physical plant’s model parameters.*

The inherent controller of the ICPS is an output feedback tracking controller formulated as

(2) $$\left\{\begin{array}{c} x_{c}\left(k+1\right)=A_{c}x_{c}\left(k\right)+B_{c}y_{e}\left(k\right),\\ u_{c}\left(k\right)=C_{c}x_{c}\left(k\right)+D_{c}y_{e}\left(k\right), \end{array}\right.$$

where $y_{e}\left(k\right)≜y_{c}\left(k\right)-y_{r}\left(k\right)$ denotes the output tracking error, with $y_{r}\left(k\right)$ being the desired output trajectory. The variables $y_{c}\left(k\right)$ and $u_{c}\left(k\right)$ represent the physical layer output received by the control layer and the control input to be sent to the physical layer, respectively.

### 2.2. Integrity and Availability Attack Model

Suppose the attacker injects integrity and availability attacks into the network layer according to the model expressed as

(3) $$\left\{\begin{array}{c} u_{p}\left(k\right)=\Phi_{a}\left(k\right)u_{c}\left(k\right),\\ y_{c}\left(k\right)=\Phi_{s}\left(k\right)y_{p}\left(k\right), \end{array}\right.$$

where $\Phi_{a}\left(k\right)$ and $\Phi_{s}\left(k\right)$ denote the time-varying attack matrices on the actuator and sensor channels, respectively. For an ideal communication network, when no attack is initiated, the attack matrices are identity matrices, meaning $u_{p}\left(k\right)=u_{c}\left(k\right)$ and $y_{c}\left(k\right)=y_{p}\left(k\right)$.

To provide a clearer physical interpretation, the multiplicative attack framework adopted herein reflects realistic operational vulnerabilities in networked industrial environments. Specifically, the actuator attack matrix $\Phi_{a}\left(k\right)$ characterizes malicious gain modifications or command corruptions introduced by compromised field control units or malicious firmware inside programmable logic controllers. Simultaneously, the sensor attack matrix $\Phi_{s}\left(k\right)$ represents sophisticated data spoofing and bias manipulations executed within vulnerable communication links or remote telemetry units. These adversarial models directly correspond to practical attack scenarios such as stealthy firmware tampering in industrial servo drives and covert measurement corruption in distributed water distribution networks.

Regarding the specific forms of the integrity and availability attacks, the following conditions are assumed.

**Assumption** **3.** *The attack space formulated by the attacker contains four specific types of attacks. These include DoS, FDI satisfying essential stealthiness, FDI stealthy against the $\chi^{2}$ detector denoted as FDI-I, and FDI stealthy against the KLD detector denoted as FDI-II. The specific real-time attack type chosen by the attacker is unknown to the defender.*

To facilitate the study of the stealthy attack types FDI-I and FDI-II, which inherently assume system observability, we add the following general assumption for the physical system defined as Equation (1).

**Assumption** **4.** *The physical system defined in Equation* (1) *is state-observable. Furthermore, valid $\chi^{2}$ and KLD detectors are assumed to exist for this system if the model parameters were known.*

To prevent attacks from causing severe damage to the physical system, the defender intends to deploy a security detector in the control layer, as shown in Figure 1. The alarm signal output by this detector will trigger preset isolation and protection mechanisms to secure the ICPS. The primary objective of this paper is to study a data-driven security detection method capable of handling the aforementioned attacks under Assumptions 1, 2, and 4.

## 3. Data-Driven Detection Mechanisms Based on DGM Quantification

**Figure 2:**
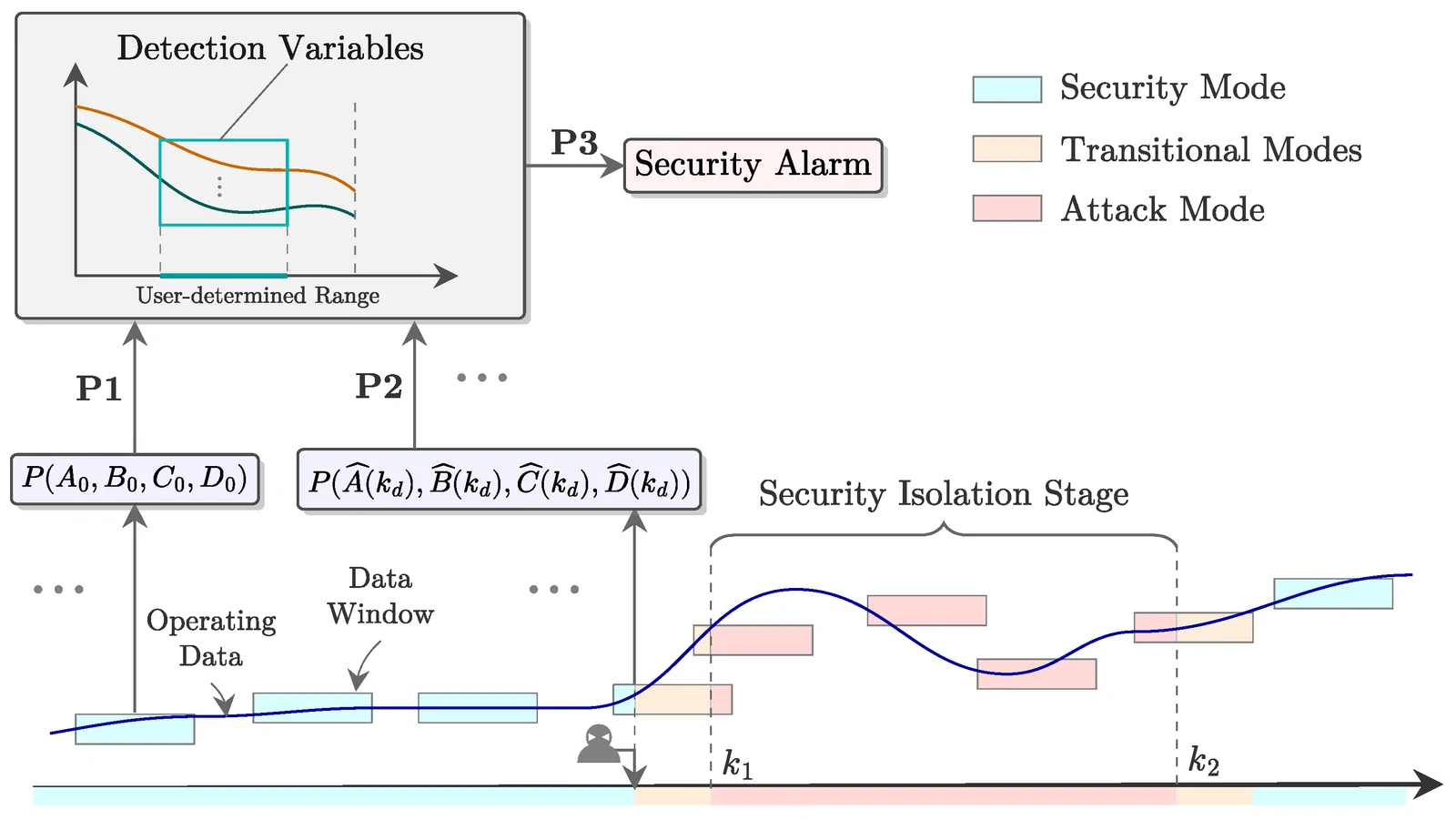
Schematic diagram of the security detection framework.

To overcome the inherent limitations of traditional model-dependent anomaly detectors heavily relying on the exact knowledge of system matrices $A_{p}$, $B_{p}$, and $C_{p}$, this section details a comprehensive data-driven detection framework (see Figure 2). This mechanism evaluates the security status of the ICPS by quantifying the DGM using solely the historical and real-time operational data collected at the control layer.

In Figure 2, the cyan, yellow, and pink boxes represent the operational data windows under security, transitional, and attack modes, respectively. P1, P2, and P3 denote the three main phases of the framework: baseline acquisition, online detection variable computation, and security alarm generation. In addition to the basic system parameters defined previously, the newly introduced symbols related to the data-driven detection methods in this section are listed in Table 2.

### 3.1. Overall Detection Strategy and Output Estimation

When the ICPS is subjected to an integrity and availability attack, the adversarial interventions fundamentally alter the generalized system model comprising the physical plant and the compromised network layer. Specifically, substituting the attack model into the nominal plant yields the compromised generalized model defined by

(4) $$\left\{\begin{array}{c} x\left(k+1\right)=A_{p}x\left(k\right)+B_{p}\Phi_{a}\left(k\right)u_{c}\left(k\right)+w_{x}\left(k\right)\\ y_{c}\left(k\right)=\Phi_{s}\left(k\right)C_{p}x\left(k\right)+\Phi_{s}\left(k\right)w_{y}\left(k\right) \end{array}\right.$$

where the adversarial matrices $\Phi_{a}\left(k\right)$ and $\Phi_{s}\left(k\right)$ force the generalized control and measurement matrices to drift from their nominal states.

In conventional control systems, state estimation residuals are widely adopted for anomaly detection. A standard Kalman filter estimates the system state $\hat{x}\left(k\right)$ using the innovation gain K(k) and relies on the precise mathematical models to generate the residual signal $z\left(k\right)=y_{c}\left(k\right)-C_{p}\hat{x}\left(k\right)$. However, this conventional paradigm becomes invalid under our established assumptions, where model parameters remain entirely unknown.

To bridge this gap, we transform the state estimation problem into a generalized parameter estimation framework. For the unknown generalized system, we consider an input–output subsystem parameterized by an unknown vector θ and a regression vector φ(k) containing historical inputs and outputs. During the secure operation mode, a recursive data-driven output estimator is constructed to iteratively approximate the subsystem dynamics. The detailed mathematical derivations and the specific iterative equations for this estimator, which is designed to minimize a predefined cost function utilizing historical operational data, have been comprehensively established in our preliminary work [25]. Consequently, the redundant formula expansions are omitted here.

The estimated parameter converges to a baseline value ${\hat{\theta }}_{0}$ at a specific steady-state instance $k_{0}$. The corresponding baseline estimation error $v_{0}=y_{c}\left(k_{0}\right)-\varphi^{⊤}\left(k_{0}\right){\hat{\theta }}_{0}$ serves as the secure threshold metric.

When attacks trigger structural mismatches in the generalized model, the real-time estimation error v(k) will deviate from its secure baseline $v_{0}$. To formalize this DGM strategy and enhance the detection sensitivity against sophisticated stealthy attacks, we present two specific subspace-based quantification methods in the following subsections encompassing static quantification via innovation sequences and dynamic quantification via extended Markov matrices.

The unified anomaly detector is evaluated using a weighted Euclidean distance metric formulated as

(5) $$\alpha \left(k\right)={∥v\left(k\right)-v_{0}∥}_{Q}^{2}\underset{S_{0}}{\overset{S_{1}}{≷}}\alpha_{th}$$

where v(k) is the real-time detection variable constructed from the subspace data; *Q* is a positive definite weighting matrix; $S_{0}$ and $S_{1}$ correspond to the secure and alarm states, respectively; and $\alpha_{th}$ is a predefined threshold configured based on an acceptable false alarm rate $\beta^{*}$.

Regarding the detailed estimation of the threshold $\alpha_{th}$ in Equation (5), it is computed empirically from a finite set of secure operational data collected during the baseline acquisition phase. Given a finite secure monitoring window of length *N*, the empirical maximum upper bound of the background fluctuations is recorded. The acceptable false-alarm rate $\beta^{*}$ is specified as a design parameter based on industrial tolerance limits, which determines the specific empirical safety margin added above the maximum bound. When the industrial plant undergoes significant operating-condition changes or shifts its reference trajectories, the nominal baseline and threshold require a brief recalibration during the transitional mode to maintain robust false-alarm resistance.

### 3.2. Static Quantification via Innovation Sequence Estimation

To mathematically formulate the subspace-based detection variables, we first introduce several fundamental definitions regarding long time-series data matrices and orthogonal projections.

**Definition** **1.** *For a long time-series data (denoted by LTD) matrix with a sequence length l, the mathematical correlation is defined by the LTD expectation expressed as*

(6) $${\bar{E}}_{l}\left[XY^{⊤}\right]≜\lim_{l\to \infty }\frac{1}{l}XY^{⊤}$$

*where the matrices X and Y are block-Hankel matrices constructed from random variables over l sampling instances.*

**Definition** **2.** *For two matrices M and N with compatible dimensions, the orthogonal projection of the row space of M onto the row space of N is denoted by M/N and computed by*

(7) $$M/N≜MN^{⊤}{\left(NN^{⊤}\right)}^{†}N$$

*where the superscript* † *represents the Moore–Penrose pseudo-inverse.*

**Definition** **3.** *To guarantee numerical stability in practical ICPS setups and circumvent the severe truncation errors caused by the pseudo-inverse operation on massive data matrices, the orthogonal projection is strictly implemented via QR decomposition. Let the augmented matrix of N and M be decomposed as*

(8) $$\left(\begin{array}{c} N\\ M \end{array}\right)=\left[\begin{array}{cc} R_{11} & 0\\ R_{21} & R_{22} \end{array}\right]\left(\begin{array}{c} Q_{1}^{⊤}\\ Q_{2}^{⊤} \end{array}\right)$$

*where $Q_{1}$ and $Q_{2}$ are orthogonal matrices and $R_{11}$ is a lower triangular matrix. The orthogonal projection is thereby robustly obtained by evaluating $M/N=R_{21}R_{11}^{-1}N$.*

Building upon these definitions, we construct the subspace equations. Assuming the physical plant is observable according to Assumption 4, there exists a stabilizing observer gain *L* rendering the matrix $A_{o}≜A_{p}-LC_{p}$ strictly Schur stable. Under secure conditions where the attack matrices equal the identity matrix *I*, the system transforms into its innovation form described by

(9) $$\left\{\begin{array}{c} x\left(k+1\right)=A_{o}x\left(k\right)+B_{p}u_{c}\left(k\right)+Ly_{c}\left(k\right)\\ y_{c}\left(k\right)=C_{p}x\left(k\right)+z\left(k\right) \end{array}\right.$$

where x(k) denotes the unmeasurable state vector and $z\left(k\right)≜y_{c}\left(k\right)-C_{p}x\left(k\right)$ denotes the innovation sequence.

We define a sliding data window of length *s*. Using the operational data sequences $u_{c}\left(k\right)$ and $y_{c}\left(k\right)$, we formulate the block-Hankel matrices $U_{f}$, $Y_{f}$, $U_{b}$, and $Y_{b}$. Each block-Hankel matrix features *p* block rows and *q* columns satisfying the dimension constraint q+2p−1=s. The subscripts *f* and *b* separate the data horizon into the past and the recent blocks, respectively.

**Theorem** **1.** *Given the control layer operational data sequences $u_{c}\left(k\right)$ and $y_{c}\left(k\right)$ of length s, a consistent estimate of the innovation sequence Z(k)={z(k−p+1),⋯,z(k−p+q+2)} corresponding to the recent data window can be obtained by*

(10) $${\hat{Z}}_{b}^{-}=Y_{b}^{-}-Y_{b}^{-}/W_{f}$$

*where $W_{f}≜{\left[U_{f}^{⊤},Y_{f}^{⊤}\right]}^{⊤}$, and the block-Hankel matrices $Y_{f}$ and $Y_{b}$ are, respectively, given by*

(11) $$Y_{f}=\left[\begin{array}{cccc} y_{c}\left(0\right) & y_{c}\left(1\right) & \cdots & y_{c}\left(q-1\right)\\ y_{c}\left(1\right) & y_{c}\left(2\right) & \cdots & y_{c}\left(q\right)\\ \vdots & \vdots & \ddots & \vdots \\ y_{c}\left(p-1\right) & y_{c}\left(p\right) & \cdots & y_{c}\left(p+q-2\right) \end{array}\right],$$

(12) $$Y_{b}=\left[\begin{array}{cccc} y_{c}\left(p\right) & y_{c}\left(p+1\right) & \cdots & y_{c}\left(p+q-1\right)\\ y_{c}\left(p+1\right) & y_{c}\left(p+2\right) & \cdots & y_{c}\left(p+q\right)\\ \vdots & \vdots & \ddots & \vdots \\ y_{c}\left(2p-1\right) & y_{c}\left(2p\right) & \cdots & y_{c}\left(2p+q-2\right) \end{array}\right].$$

*The number of block rows satisfies $n_{x}<p\ll s$, and the number of columns q satisfies q=s−2p+1, with $y_{c}\left(0\right)≜y_{c}\left(k-s+1\right),\ldots ,y_{c}\left(2p+q-2\right)≜y_{c}\left(k\right)$. The structures of $U_{f}$ and $U_{b}$ are identical to those of $Y_{f}$ and $Y_{b}$.*

**Proof.**  Based on the innovation model defined previously and the data sequences, the subspace equations can be formulated by recursive expansion

(13) $$\begin{array}{cc} Y_{f} & =\Gamma X_{f}+H_{u}U_{f}+H_{y}Y_{f}+Z_{f},\\ Y_{b} & =\Gamma X_{b}+H_{u}U_{b}+H_{y}Y_{b}+Z_{b},\\ X_{b} & =A_{o}^{p}X_{f}+F_{u}U_{f}+F_{y}Y_{f}. \end{array}$$

In Equation (13), $Z_{f}$ and $Z_{b}$ are block-Hankel matrices constructed from the innovation sequence z(k). The augmented state variables $X_{f}$ and $X_{b}$ and the extended observability matrix Γ are, respectively, expressed as

(14) $$X_{f}=\left[\begin{array}{cccc} x(0) & x(1) & \cdots & x(q-1) \end{array}\right],$$

(15) $$X_{b}=\left[\begin{array}{cccc} x(p) & x(p+1) & \cdots & x(p+q-1) \end{array}\right],$$

(16) $$\Gamma ={\left[\begin{array}{ccccc} C_{p}^{⊤} & {\left(C_{p}A_{o}\right)}^{⊤} & {\left(C_{p}A_{o}^{2}\right)}^{⊤} & \cdots & {\left(C_{p}A_{o}^{p-1}\right)}^{⊤} \end{array}\right]}^{⊤}.$$

$H_{u}$ and $H_{y}$ are lower triangular block-Toeplitz matrices with identical structures. Taking $H_{u}$ as an example

(17) $$H_{u}=\left[\begin{array}{ccccc} 0 & 0 & 0 & \cdots & 0\\ C_{p}B_{p} & 0 & 0 & \cdots & 0\\ C_{p}A_{o}B_{p} & C_{p}B_{p} & 0 & \cdots & 0\\ \vdots & \vdots & \vdots & \ddots & \vdots \\ C_{p}A_{o}^{p-2}B_{p} & C_{p}A_{o}^{p-3}B_{p} & C_{p}A_{o}^{p-4}B_{p} & \cdots & 0 \end{array}\right].$$

$F_{u}$ and $F_{y}$ also share identical structures, as shown by

(18) $$F_{u}=\left[\begin{array}{ccccc} A_{o}^{p-1}B_{p} & A_{o}^{p-2}B_{p} & \cdots & A_{o}B_{p} & B_{p} \end{array}\right].$$

Consider the subspace state equation in Equation (13). Since the system is observable and $A_{o}=A_{p}-LC_{p}$ is strictly stable, for a sufficiently large *p*, we have

(19) $$\begin{array}{cc} {\bar{E}}_{p}\left[X_{b}\right] & ={\bar{E}}_{p}\left[A_{o}^{p}X_{f}+F_{u}U_{f}+F_{y}Y_{f}\right]\\ \; & ={\bar{E}}_{p}\left[F_{u}U_{f}+F_{y}Y_{f}\right]. \end{array}$$

Denoting ${\hat{X}}_{b}=F_{u}U_{f}+F_{y}Y_{f}$, $H≜H_{u}U_{b}+H_{y}Y_{b}+Z_{b}$ and substituting it into the expression of $Y_{b}$ in Equation (13) to eliminate $X_{b}$, we obtain

(20) $$\hat{\Gamma }\left(F_{u}U_{f}+F_{y}Y_{f}\right)=Y_{b}-\hat{H},$$

and the corresponding estimation error is ${\bar{E}}_{p}\left[\Gamma A_{o}^{p}X_{f}\right]=0$.

Extracting the first block row of Equation (20) (denoted by the superscript “−”) and noting that the first block rows of the strictly lower triangular block-Toeplitz matrices $H_{u}$ and $H_{y}$ are identically zero matrices (i.e., $H_{u}^{-}=0$ and $H_{y}^{-}=0$), we deduce

(21) $${\hat{C}}_{p}\left(F_{u}U_{f}+F_{y}Y_{f}\right)=Y_{b}^{-}-{\hat{Z}}_{b}^{-},$$

where $Y_{b}^{-}$ and $Z_{b}^{-}$ denote the first block rows of the matrices $Y_{b}$ and $Z_{b}$ (i.e., the first $n_{y}$ rows), respectively.

Defining the combined past data matrix $W_{f}={\left[U_{f}^{⊤},Y_{f}^{⊤}\right]}^{⊤}$, Equation (21) can be rewritten as

(22) $$Y_{b}^{-}={\hat{C}}_{p}\left[\begin{array}{cc} F_{u} & F_{y} \end{array}\right]W_{f}+{\hat{Z}}_{b}^{-}.$$

Applying the orthogonal projection operation onto the row space of $W_{f}$ on both sides of Equation (22) yields

(23) $$Y_{b}^{-}/W_{f}={\hat{C}}_{p}\left[\begin{array}{cc} F_{u} & F_{y} \end{array}\right]W_{f}+{\hat{Z}}_{b}^{-}/W_{f}.$$

Crucially, the innovation sequence $Z_{b}^{-}$ behaves as white noise and is statistically uncorrelated with the past data matrix $W_{f}$, meaning ${\bar{E}}_{s}\left[{\hat{Z}}_{b}^{-}/W_{f}\right]=0$. Therefore, substituting this property into Equation (23) and combining it with Equation (22), we obtain the consistent estimate of the innovation sequence

(24) $${\hat{Z}}_{b}^{-}=Y_{b}^{-}-{\hat{C}}_{p}\left[\begin{array}{cc} F_{u} & F_{y} \end{array}\right]W_{f}=Y_{b}^{-}-Y_{b}^{-}/W_{f}.$$

The estimation error of ${\hat{Z}}_{b}^{-}$ is ${\bar{E}}_{s}\left[{\hat{Z}}_{b}^{-}/W_{f}\right]=0$. Since $Z_{b}^{-}$ consists of the first $n_{y}$ rows of $Z_{b}$, according to Equation (12), for the operational data generated from time k−s+1 to time *k*, we have $Z_{b}^{-}=Z\left(k\right)=\left\{z\left(k-p+1\right),\cdots ,z\left(k-p+q+2\right)\right\}$. Thus, Equation (24) is a consistent estimate of the innovation sequence Z(k). This completes the proof. □

**Remark** **2.** *The core essence of Theorem 1 lies in separating the predictable system baseline from unpredictable anomalies using pure data-driven orthogonal projections. By projecting the recent operational data onto the mathematical subspace spanned by the historical observation data, the predictable functional relationships are effectively isolated. The remaining orthogonal residual directly constitutes the innovation sequence representing the novel information or potential disturbances entering the physical plant. Building upon this theoretical decoupling mechanism, the estimated innovation vector transformed into a column sequence $v_{s}\left(k\right)≜\mathrm{vec}\left({\hat{Z}}_{b}^{-}\right)$ serves as the primary static detection variable. Any numerical deviations observed in $v_{s}\left(k\right)$ relative to its nominal secure baseline $v_{s,0}$ effectively capture the steady-state discrepancies imposed by essential attacks.*

**Remark** **3.** *It is worth noting from a finite-sample perspective that because the subspace matrices are constructed using finite operational data rather than infinite realizations, the empirical estimation error of the innovation sequence is bounded by the persistent excitation level of the input data and the singular value distribution of the Hankel matrices. As the data window sample size increases, the empirical distribution of the finite-sample estimation converges reliably to its theoretical counterpart.*

### 3.3. Dynamic Quantification via Extended Markov Matrix Estimation

While the static innovation sequence strictly evaluates the amplitude variations representing the steady-state model mismatch, capturing the temporal transition behaviors is universally required for mitigating advanced stealthy anomalies. To accomplish dynamic quantification, we leverage the Markov parameters of the generalized system.

**Definition** **4.** *For a typical discrete LTI system governed by matrices A, B, C, and D, the Markov parameter sequence denoted by J(p) represents the impulse response data over p sampling steps formulated as $J\left(p\right)=\left\{D,CB,CAB,\ldots ,CA^{p-2}B\right\}$. The matrix constructed sequentially by these block elements constitutes the complete Markov matrix identifying the dynamic transient properties of the system.*

**Theorem** **2.** *For the ICPS consisting of the physical plant and the tracking controller, and given the control layer operational data sequences $u_{c}\left(k\right)$ and $y_{c}\left(k\right)$ of length s, when the system input satisfies the persistent excitation condition, a consistent estimate of the extended Markov matrix $M=C_{p}\left[\begin{array}{cc} F_{u} & F_{y} \end{array}\right]$ from the augmented input $\zeta \left(k\right)≜{\left[u_{c}^{⊤}\left(k\right),y_{c}^{⊤}\left(k\right)\right]}^{⊤}$ to the system output $y_{c}\left(k+1\right)$ is given by*

(25) $$M\left(k\right)=Y_{b}^{-}W_{f}^{⊤}{\left(W_{f}W_{f}^{⊤}\right)}^{-1}$$

**Proof.**  The rigorous mathematical derivations regarding the consistent estimation of the extended Markov matrix have been presented in our preliminary work [26]. The process primarily relies on the intermediate algebraic result $Y_{b}^{-}/W_{f}=MW_{f}$ derived from the subspace projection operations. When the control inputs fulfill the persistent excitation condition, the unique optimal solution can be obtained by applying the standard least squares estimation method, which results in Equation (25). This completes the proof. □

**Remark** **4.** *Theorem 2 further advances the static security evaluation by providing a real-time consistent estimator for the dynamic matrix features. This theorem mathematically guarantees that even under severe unknown measurement noises, the inherent dynamic characteristics of the physical plant can be accurately tracked through the block-Hankel matrices. The calculated extended Markov matrix continuously reflects the authentic dynamic mapping relationship between the inputs and outputs. Consequently, the dynamic detection variable is subsequently constructed using the vectorization operation, resulting in $v_{d}\left(k\right)≜\mathrm{vec}\left(\hat{M}\left(k\right)\right)$. Substituting $v_{d}\left(k\right)$ back into the generalized distance metric framework expressed in Equation* (5) *fully establishes the dynamic anomaly detector targeting sophisticated temporal manipulations.*

### 3.4. Geometric Mechanism Analysis

To better understand why certain detection variables are effective against advanced stealthy attacks while others fail, it is necessary to analyze the geometric mechanism of the proposed DGM method in the subspace domain.

**Figure 3:**
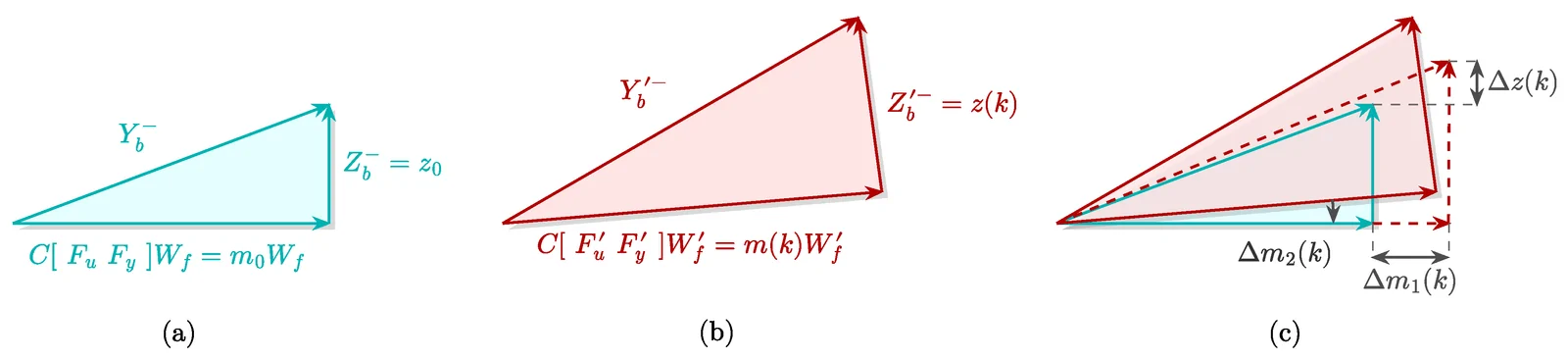
Geometric interpretations of two types of detection variables. (**a**) The orthogonal relationship between the past data $W_{f}$ and the innovation $Z_{b}^{-}$ under secure conditions. (**b**) The subspace distortion under an integrity and availability attack. (**c**) Comparison of deviations in the static and dynamic detection variables.

As illustrated in Figure 3a, which shows the orthogonal relationship between the past data $W_{f}$ and the innovation $Z_{b}^{-}$ under secure conditions, the row space of the past data matrix $W_{f}$ (represented horizontally) is strictly orthogonal to the row space of the innovation matrix $Z_{b}^{-}$ (represented vertically). The geometric interpretation of Theorem 1 is that the nominal output $Y_{b}^{-}$ is decomposed into two orthogonal components: the projection onto the past data subspace ($M_{0}W_{f}$) and the orthogonal residual ($Z_{b}^{-}$).

When an IA attack occurs, it alters the inherent structures of the system matrices. Geometrically, as shown in Figure 3b (illustrating the subspace distortion under an integrity and availability attack) and Figure 3c (displaying the comparison of deviations in the static and dynamic detection variables), the attack stretches and rotates the original row space $M_{0}W_{f}$ into a distorted space denoted by $M\left(k\right)W_{f}^{\prime }$. To facilitate a direct comparison between the secure and attacked states, Figure 3c aligns the two geometric representations. The dashed lines serve purely as auxiliary lines to visualize the dimensional changes in the subspace matrices. Specifically, the gray arrows denoted by Δz(k) and $\Delta m_{1}\left(k\right)$ represent the magnitude variations in the innovation sequence (the right edge) and the base subspace matrix (the bottom edge) before and after the attack, respectively. Meanwhile, the gray arrow $\Delta m_{2}\left(k\right)$ indicates the angular rotational shift of the subspace caused by the attack.

In an FDI-II attack, the attacker carefully designs the injected noise signals to maintain the orthogonal relationship between the compromised outputs and the historical data. Consequently, the rotation angle $\Delta m_{2}\left(k\right)$ of the row space is significantly restricted. This causes the variation in the innovation sequence, Δz(k), to be extremely small and often fall below the detection threshold $\alpha_{th}$. As a result, the static innovation detector fails to detect the attack.

In contrast, the extended Markov matrix $\hat{M}\left(k\right)$ evaluates both the linear stretching and rotational shifts of the subspace, represented by $\Delta m_{1}\left(k\right)$ and $\Delta m_{2}\left(k\right)$. By capturing the dynamic characteristics and generalized impulse responses of the system, this dynamic detection mechanism is highly sensitive to the structural changes caused by attacks, thereby successfully detecting stealthy attacks.

To provide a clearer intuitive illustration of the theoretical derivations and heavy subspace notations, consider a practical operational instance where the sliding data window receives continuous input–output samples from the control layer. As historical data accumulate into the block-Hankel matrices $U_{f}$ and $Y_{f}$, the subspace projection operation isolates the predictable functional relationship without requiring explicit knowledge of the physical system matrices $A_{p}$ and $B_{p}$. When a stealthy attack occurs, the data flow entering the Hankel matrices causes an immediate structural rotation, which is quantitatively captured by the projection residual or the extended Markov matrix estimator. This step-by-step data processing workflow ensures that abstract mathematical formulations are directly translated into intuitive, real-time security indicators.

## 4. Hardware Experiments and Analysis of Results

Before delving into the hardware implementation, it is worth noting that the preliminary validation of the proposed data-driven detection methodology has been conducted through numerical simulations on a Western States Coordinated Council (WSCC) 9-bus power system, which is detailed in our previous work [25]. Building upon those fundamental simulation results, this section is dedicated to further evaluating the practical performance and robustness of the proposed detectors against sophisticated stealthy attacks using a physical testbed.

In this section, a two degree-of-freedom (2-DoF) robot and a DC servo motor (both manufactured by Quanser Inc., Markham, ON, Canada) are selected as the controlled physical plants of the ICPS to experimentally validate and evaluate the performance of the two detection methods proposed in Section 3. The experiments are divided into two parts. The first experiment aims to verify the detection performance of the two detectors against DoS and essential FDI attacks, respectively. The second experiment is designed to evaluate their performance against the first and second types of stealthy FDI attacks (i.e., FDI-I and FDI-II).

**Figure 4:**
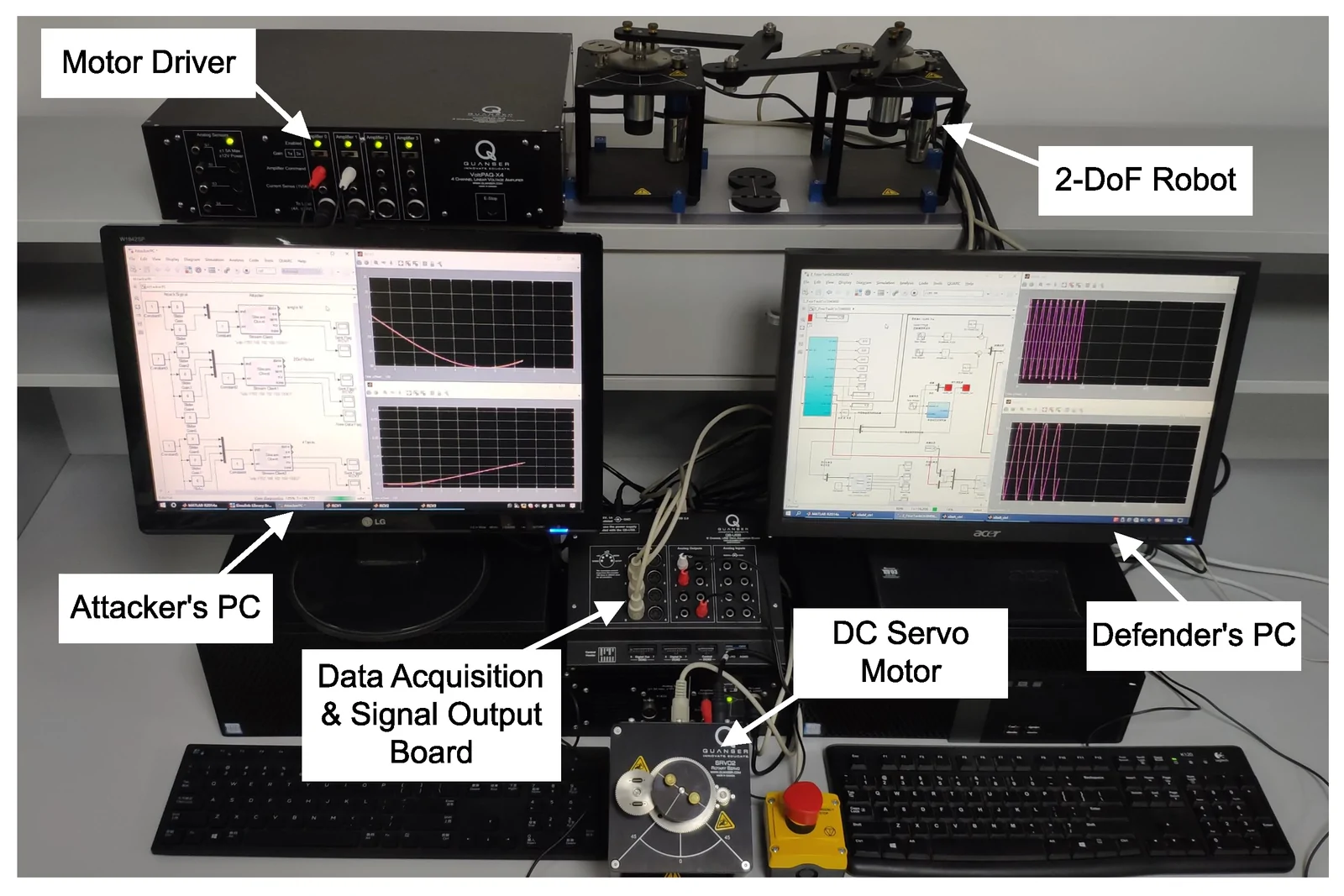
The HIL experimental platform for 2-DoF robots and a servo motor.

The established hardware-in-the-loop (HIL) experimental platform is shown in Figure 4. The defender’s controller and detector are deployed in a dedicated HIL environment (Quarc 2019 SP1, Quanser Inc., Markham, ON, Canada) on the defender’s PC, while the attacker implements the four attack strategies based on the HIL environment on another PC to simulate practical IA attack scenarios. UDP communication protocol is used for data exchange between the two PCs to construct the network layer of the ICPS.

**Figure 5:**
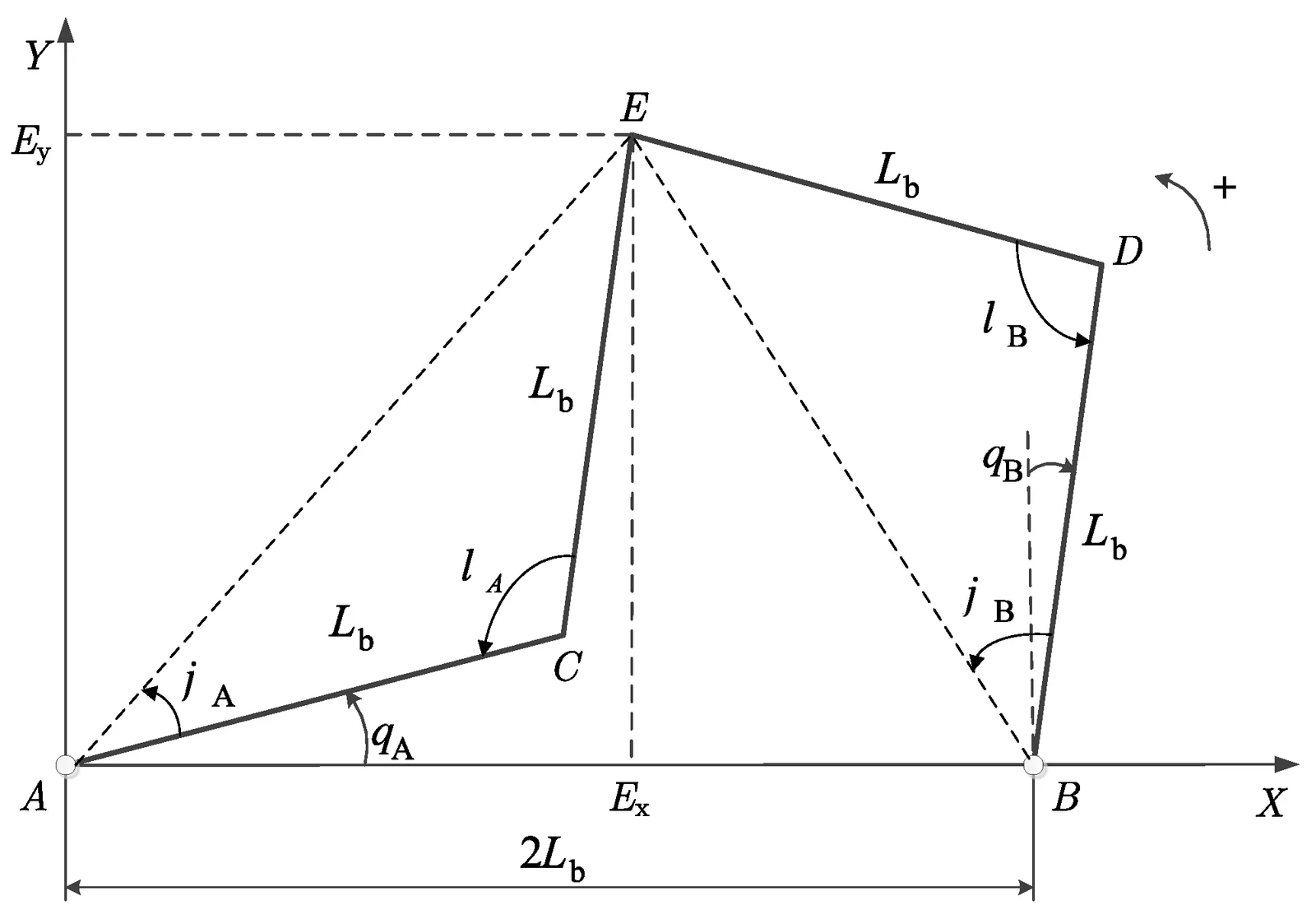
Linkage mechanism of the 2-DoF robot.

**Figure 6:**
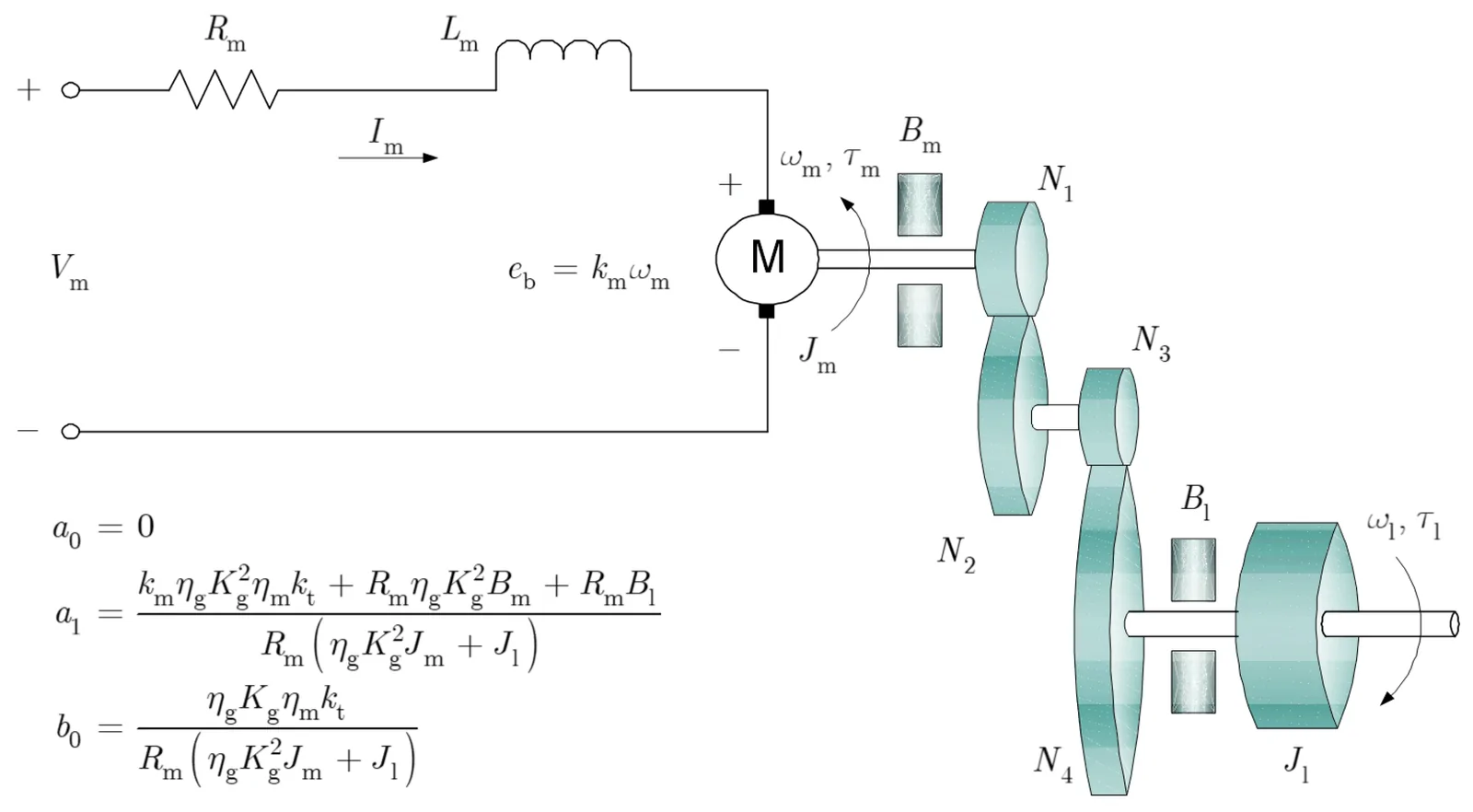
The DC servo motor armature circuit and its driven components.

The 2-DoF robot consists of two DC servo motors and a rigid four-bar linkage mechanism, as shown in Figure 5. Here, points A, B, C, and *D* denote the rotational joints, and point *E* is the end-effector. $L_{b}$ represents the length of the rigid links, while $q_{A}$ and $q_{B}$ indicate the rotational angles controlled by the two servo motors. The end-effector is point *E*, and the control objective is to make point *E* track a predefined trajectory. The servo motor in the robot system has the same structure as the standalone servo motor module, and its armature circuit and gear transmission components are shown in Figure 6. The output of the standalone servo motor system is the motor rotation angle, and the objective is angle tracking control. The inherent controllers for the systems are all discrete PID controllers. The desired trajectory coordinates for the end-effector *E* of the 2-DoF robot are defined as $y_{r}\left(k\right)≜{\left[y_{rx}\left(k\right),y_{ry}\left(k\right)\right]}^{⊤}$, where $y_{rx}\left(k\right)=5+\sin\left(0.05\pi k\right)$ and $y_{ry}\left(k\right)=5+\sin\left(0.1\pi k\right)$. The desired output angle for the servo motor module is $y_{rm}\left(k\right)=10\sin\left(0.1\pi k\right)$.

### 4.1. Performance Evaluation Under DoS and Essential FDI Attacks

The purpose of this experiment is to verify the detection performance of the innovation sequence estimator and the extended Markov detector against DoS and essential FDI attacks, respectively. HIL experiments are conducted on the established platform with a sampling time of 0.02 s and a total experimental duration of 200 s. The attacker launches DoS and essential FDI attacks against the servo motor and the 2-DoF robot subsystems, respectively, starting from 100 s until the end of the experiment. The two proposed detectors are deployed in the control layer to monitor the security status of the ICPS.

Regarding the attack parameter settings, the DoS attack targets both the sensor and actuator channels of the servo motor subsystem, and the corresponding packet loss probabilities are both set to 35% to avoid hardware damage to the experimental system. The parameters for the essential FDI attack are set as shown in Equation (26):

(26) $$\left\{\begin{array}{c} \Phi_{a}\left(k\right)=0.0167k^{\prime }\mathrm{diag}\left\{1,1.2\right\},\\ \Phi_{s}\left(k\right)=I+S\left(k\right)\mathrm{diag}{\left\{y_{p1}\left(k\right),y_{p2}\left(k\right)\right\}}^{-1},\\ S(k)=\mathrm{diag}\{-0.5+1.2\sin(0.05\pi k),0.5-1.2\sin(0.05\pi k)\}. \end{array}\right.$$

where $k^{\prime }=k-k_{0}$, with $k_{0}$ representing the attack onset time, and S(k) representing the FDI attack bias signal. In terms of the detector parameter settings, the number of block rows of the subspace matrix is set to p=5, the data window length is s=1009, the data length of the innovation sequence (i.e., the number of columns in the subspace matrix) is q=1000, and the weighting matrices *Q* for both the innovation estimation detector and the extended Markov detector are set as identity matrices.

The assignment of these specific values is jointly determined by the theoretical prerequisites of the subspace matrix processing techniques and the practical operational conditions of the physical system. Specifically, the number of block rows is chosen as five to guarantee that the extended observability matrix achieves full column rank, which is necessary to fully capture the underlying mechanical dynamics of the robot without inducing excessive computational delay. The column dimension is set to one thousand to ensure the statistical consistency of the data-driven estimation, effectively smoothing out the non-Gaussian noises inherent in the hardware sensors. The total sliding window length of one thousand and nine is subsequently dictated by the strict mathematical construction requirements of the block-Hankel matrices. Finally, the weighting matrices are configured as identity matrices to evaluate all measurement channels uniformly without introducing subjective baseline bias.

In the HIL experimental environment, the predefined detection threshold is determined empirically during the initial baseline acquisition phase to ensure robustness against false alarms. Because pure theoretical thresholds often fail to account for unmodeled mechanical friction and non-Gaussian measurement noises inherent in physical servo motors, we operate the 2-DoF robot under normal secure conditions to observe the steady-state responses. By recording the maximum fluctuation upper bound of the detection variable during this secure phase and incorporating a slight empirical margin, the threshold is decisively established. This practical calibration effectively absorbs the unmodeled systemic disturbances and forms a reliable baseline for the subsequent online security monitoring.

**Figure 7:**
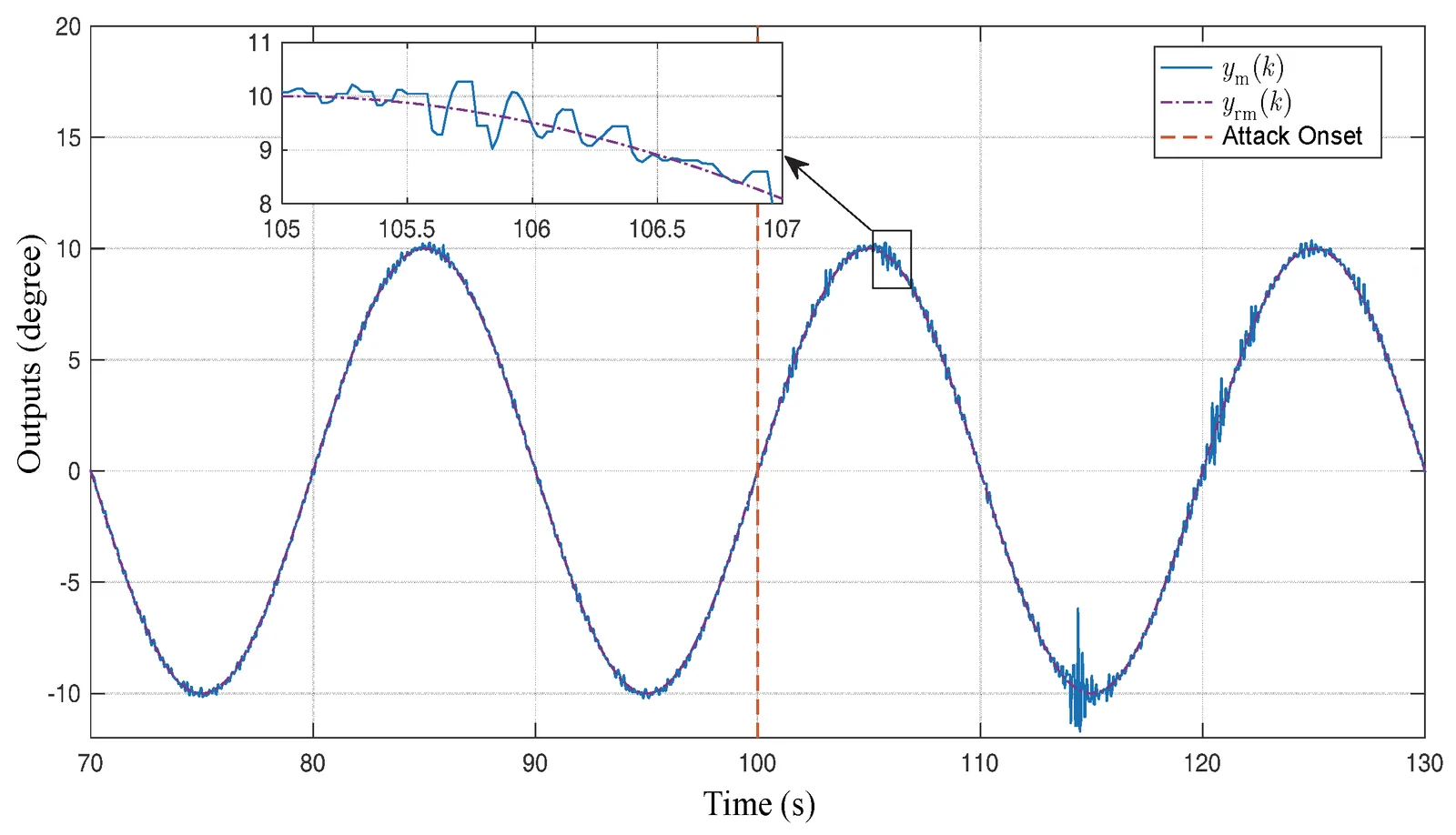
Servo motor output tracking control curve under DoS attack.

Figure 7 presents the output curves of the servo motor system under DoS attack. To highlight the changes in system tracking performance before and after the attack, only 60 s of data are shown. It can be observed that under a relatively low packet loss rate, the system can still track the predefined trajectory but with noticeable performance degradation. The reason is that both the control layer and physical layer of the system contain zero-order holds, which is common in practice. Moreover, as seen from Figure 7, the DoS attack does not satisfy essential stealthiness, but since it is stealthy to the $\chi^{2}$ detector, researching effective detection methods for this type of attack remains valuable.

**Figure 8:**
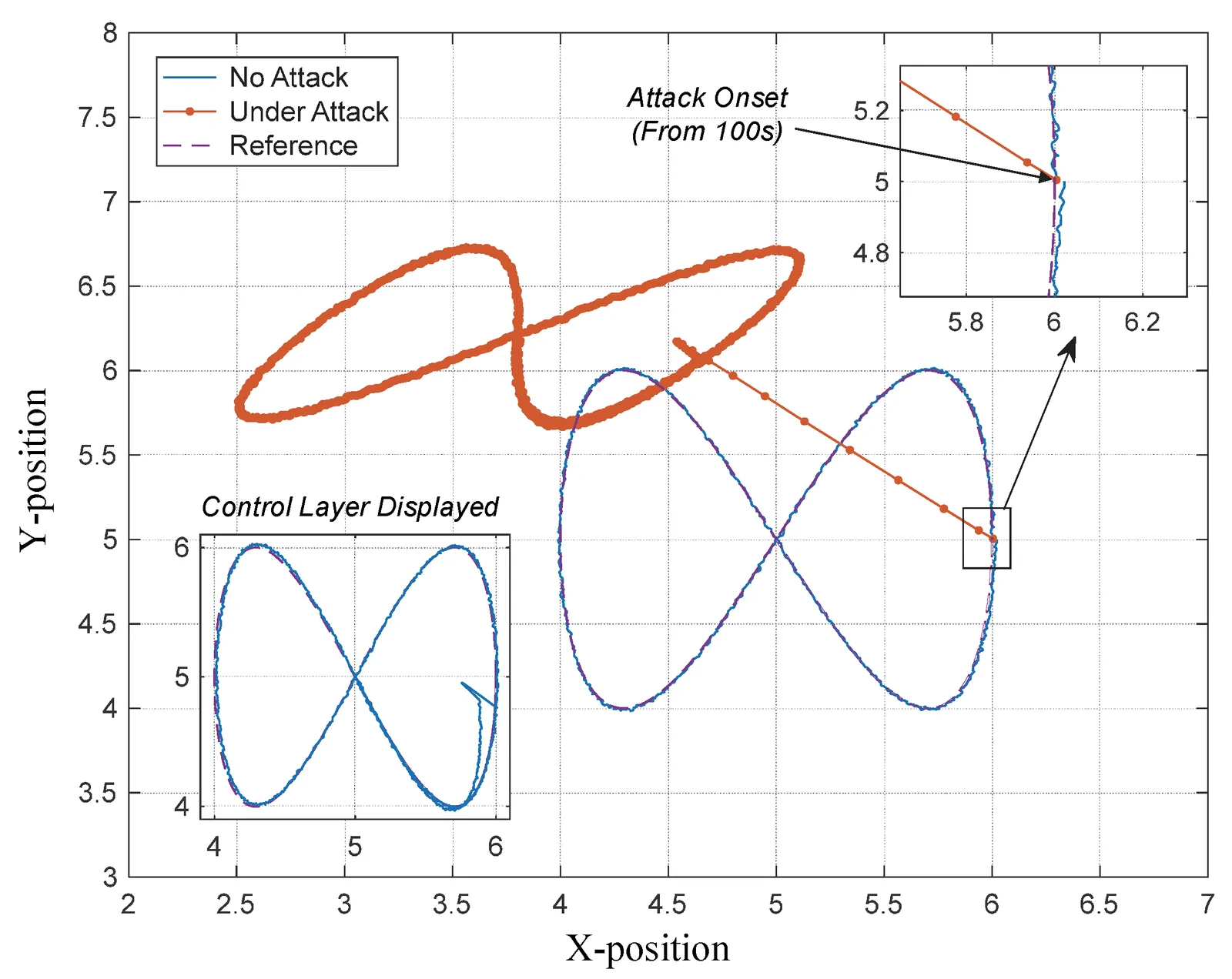
Trajectory tracking curves of the 2-DoF robot under FDI attacks.

The operating trajectory of the 2-DoF robot system under essential FDI attack is given in Figure 8. In this figure, the main central plot illustrates the actual operational trajectory of the physical plant. Here, the solid blue line represents the tracking control curve of the robot under normal secure conditions, the solid orange line with dot markers indicates the actual compromised trajectory under attack, and the dashed purple line represents the predefined reference trajectory. The inset in the upper-right corner provides a local magnified view of the main plot at the attack onset. Meanwhile, the sub-figure in the lower-left corner shows the output trajectory as displayed from the perspective of the control layer. It is clear that this control layer curve shows no obvious anomalies before and after the attack, indicating that the selected attack parameters satisfy essential stealthiness. On the other hand, the actual operating trajectory of the physical layer completely deviates from the predefined trajectory under the attack, demonstrating that the attacker has achieved the intended target.

**Figure 9:**
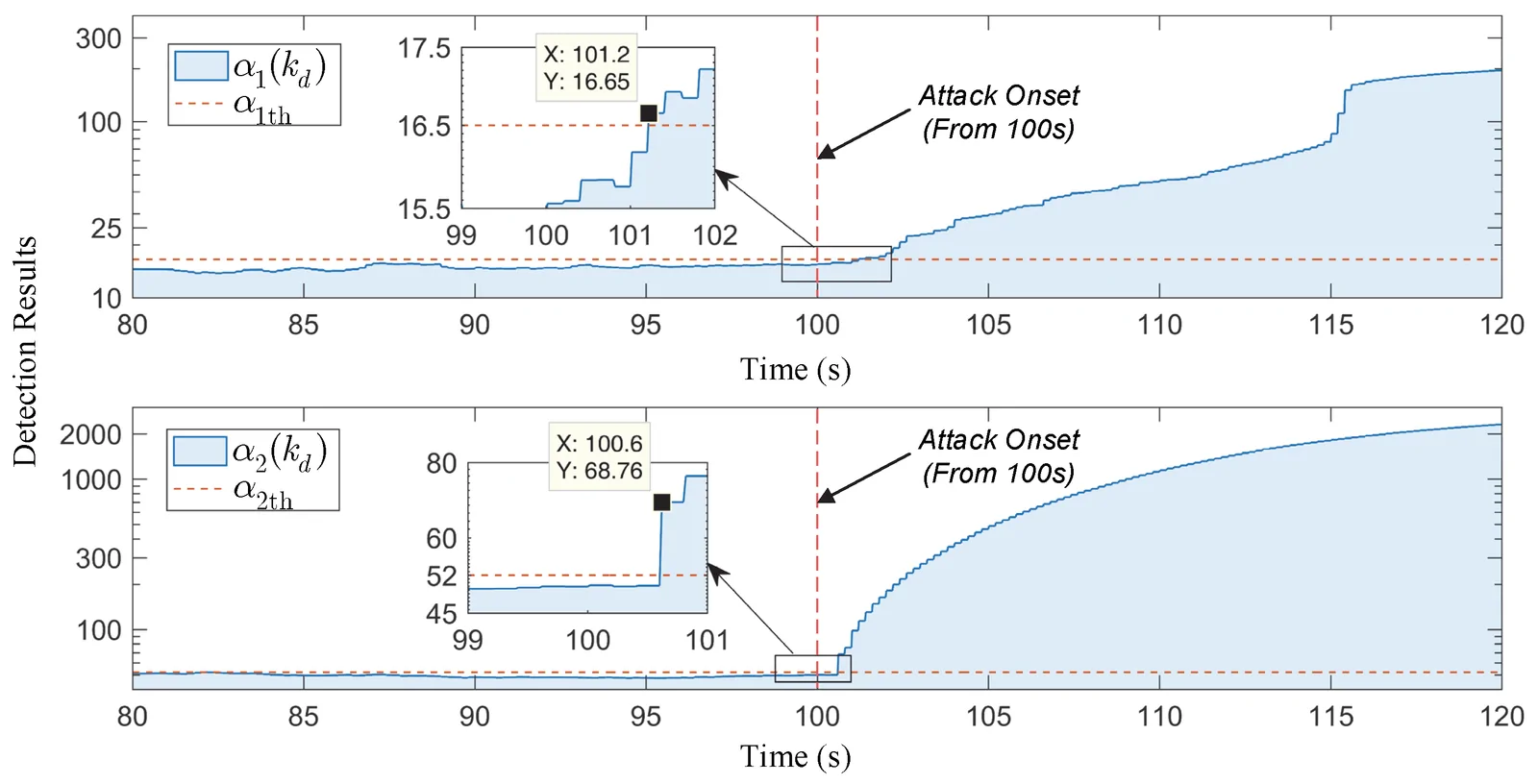
Detection results of the innovation detector for DoS and essential FDI attacks.

**Figure 10:**
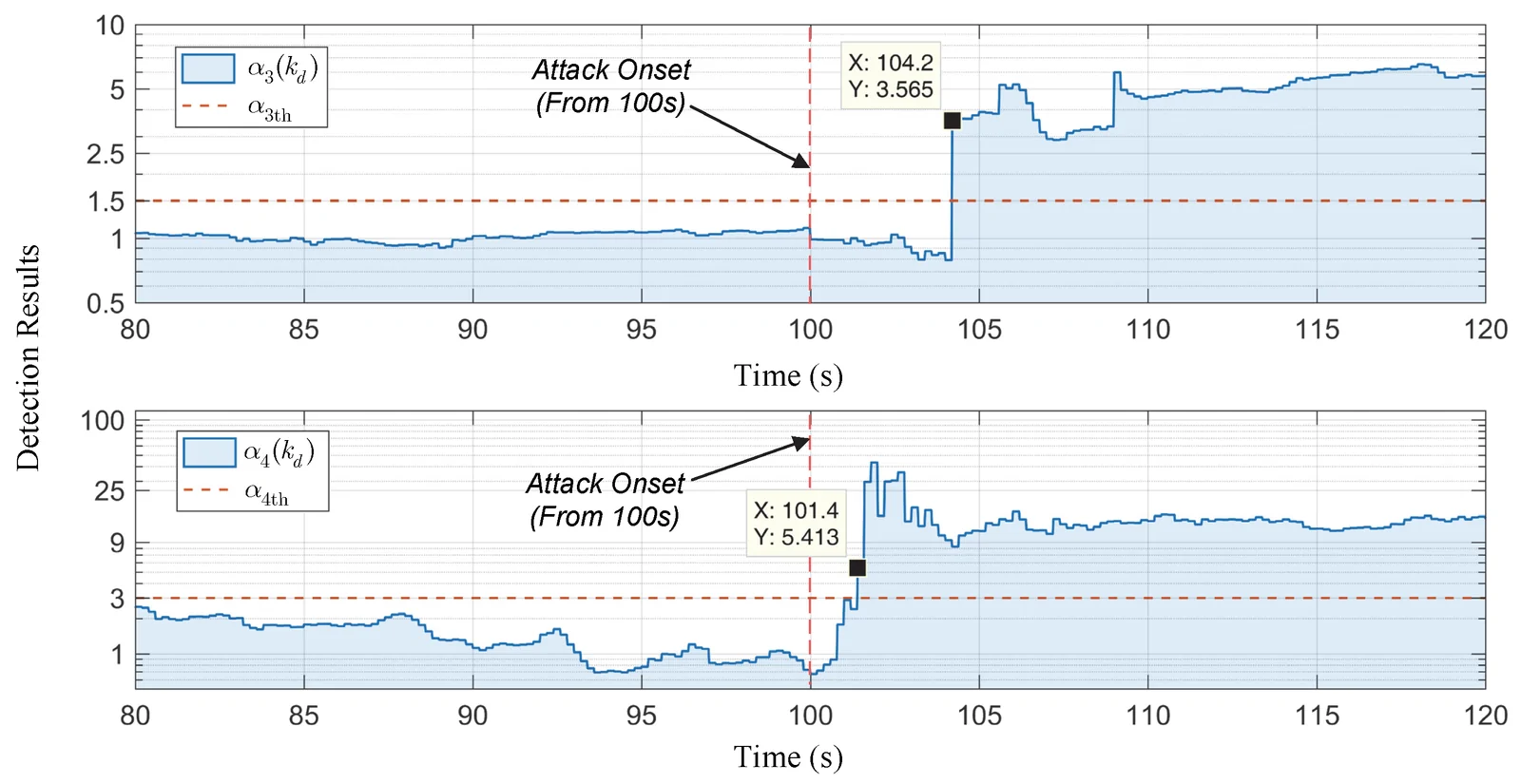
Detection results of the extended Markov detector for DoS and essential FDI attacks.

In terms of detection performance, Figure 9 and Figure 10 show the detection results of the two types of detectors against the two types of attacks, respectively, which are the main focus of this experiment. Figure 9 shows the output curves of the innovation estimation detector for DoS and essential FDI attacks, with detection thresholds $\alpha_{1th}=16.5$ and $\alpha_{2th}=52$. Note that although the three motors in the physical layer have identical parameters, the physical meanings of the outputs for the servo motor and the 2-DoF robot subsystems are different (angle vs. position), leading to distinct $\alpha_{1th}$ and $\alpha_{2th}$. The subscript $k_{d}$ in the detection variables $\alpha_{1}\left(k_{d}\right)$ and $\alpha_{2}\left(k_{d}\right)$ denotes the detection instant, meaning the detection period is set independently of the ICPS operating period; in our experiments, the detection period is uniformly set to 0.2 s. From Figure 9, it can be seen that the proposed innovation estimation detector is effective against both DoS and essential FDI attacks.

Figure 10 presents the detection results of the extended Markov detector proposed in Section 3 against DoS and essential FDI attacks, where the detection thresholds are $\alpha_{3th}=1.5$ and $\alpha_{4th}=3$, respectively. It can be seen that this detector is equally effective against both attack types.

### 4.2. Performance Evaluation Under FDI-I and FDI-II Attacks

The purpose of this experiment is to verify the detection performance of the innovation estimation detector and the extended Markov detector against two types of stealthy attacks (i.e., FDI-I and FDI-II), where FDI-I is stealthy against the $\chi^{2}$ detector and FDI-II is stealthy against the KLD detector. HIL experiments are conducted on the established platform with a sampling time of 0.02 s and an experimental duration of 200 s. The attacker initiates FDI-I and FDI-II attacks on the servo motor and 2-DoF robot subsystems, respectively, starting from the 100th second until the end of the experiment. Both proposed detectors are deployed in the control layer to monitor the ICPS security status.

Regarding the attack parameter settings, the FDI-I attack adopts the strategy shown by

(27) $$\left\{\begin{array}{c} \Phi_{a}\left(k\right)=I,\\ \Phi_{s}\left(k\right)=I+S\left(k\right)\mathrm{diag}{\left\{y_{p1}\left(k\right),y_{p2}\left(k\right)\right\}}^{-1},\\ S\left(k\right)=-C_{p}A_{o}^{k}x_{a}\left(0\right), \end{array}\right.$$

where $x_{a}\left(0\right)$ is the initial state of the attack bias signal, set as $x_{a}\left(0\right)={\left[5,5\right]}^{⊤}$. In terms of stealthiness, the attack bias signal S(k) causes identical deviations in both the output $y_{c}\left(k\right)$ received by the control layer and the output estimate $C_{p}\hat{x}\left(k\right)$ based on state observation. Therefore, the state estimation residual term remains unchanged before and after the attack, ensuring stealthiness against the $\chi^{2}$ detector. Note that the discrete parameters for the servo motor model are given by

(28) $$A_{p}=\left[\begin{array}{cc} 1 & 0.0159\\ 0 & 0.6169 \end{array}\right],\;B_{p}=\left[\begin{array}{c} 0.0063\\ 0.5863 \end{array}\right],\;C_{p}=\left[\begin{array}{cc} 1 & 0 \end{array}\right].$$

The matrix $A_{p}$ has eigenvalues 1 and 0.6169, which causes the attack bias signal to eventually converge to a constant value, thereby achieving the intended attack goal.

The parameters for the FDI-II attack are formulated as

(29) $$\left\{\begin{array}{c} \Phi_{a}\left(k\right)=\mathrm{diag}\left\{a_{1}\left(k\right),a_{2}\left(k\right)\right\},\\ \Phi_{s}\left(k\right)=I+S\left(k\right)\mathrm{diag}{\left\{y_{p1}\left(k\right),y_{p2}\left(k\right)\right\}}^{-1},\\ S\left(k\right)=z_{a}\left(k\right)+C_{p}\hat{x}\left(k\right), \end{array}\right.$$

where $a_{1}\left(k\right),a_{2}\left(k\right)∼N\left(1,0.06\right)$, and $z_{a}\left(k\right)$ is independent and identically distributed with the secure residual signal $z_{0}\left(k\right)$, generated online by the attacker. It can be seen that the actuator attack parameter $\Phi_{a}\left(k\right)$ is used to compromise the physical system’s control performance, while the sensor channel circumvents the KLD detector by generating $z_{a}\left(k\right)$ with the same distribution as $z_{0}\left(k\right)$.

It is worth noting that traditional residual-based detectors including the $\chi^{2}$ and KLD detectors are purposefully not included as control groups in the following experimental evaluations. Because the formulated FDI-I and FDI-II attack strategies mathematically guarantee the strict preservation of the statistical properties of the innovation sequences, the theoretical failure of these traditional detectors is a mathematical certainty rather than an empirical hypothesis. Consequently, displaying their non-responsive detection curves would yield redundant information. The experimental evaluations herein focus strictly on demonstrating how the proposed DGM framework successfully breaks this theoretical undetectability.

**Figure 11:**
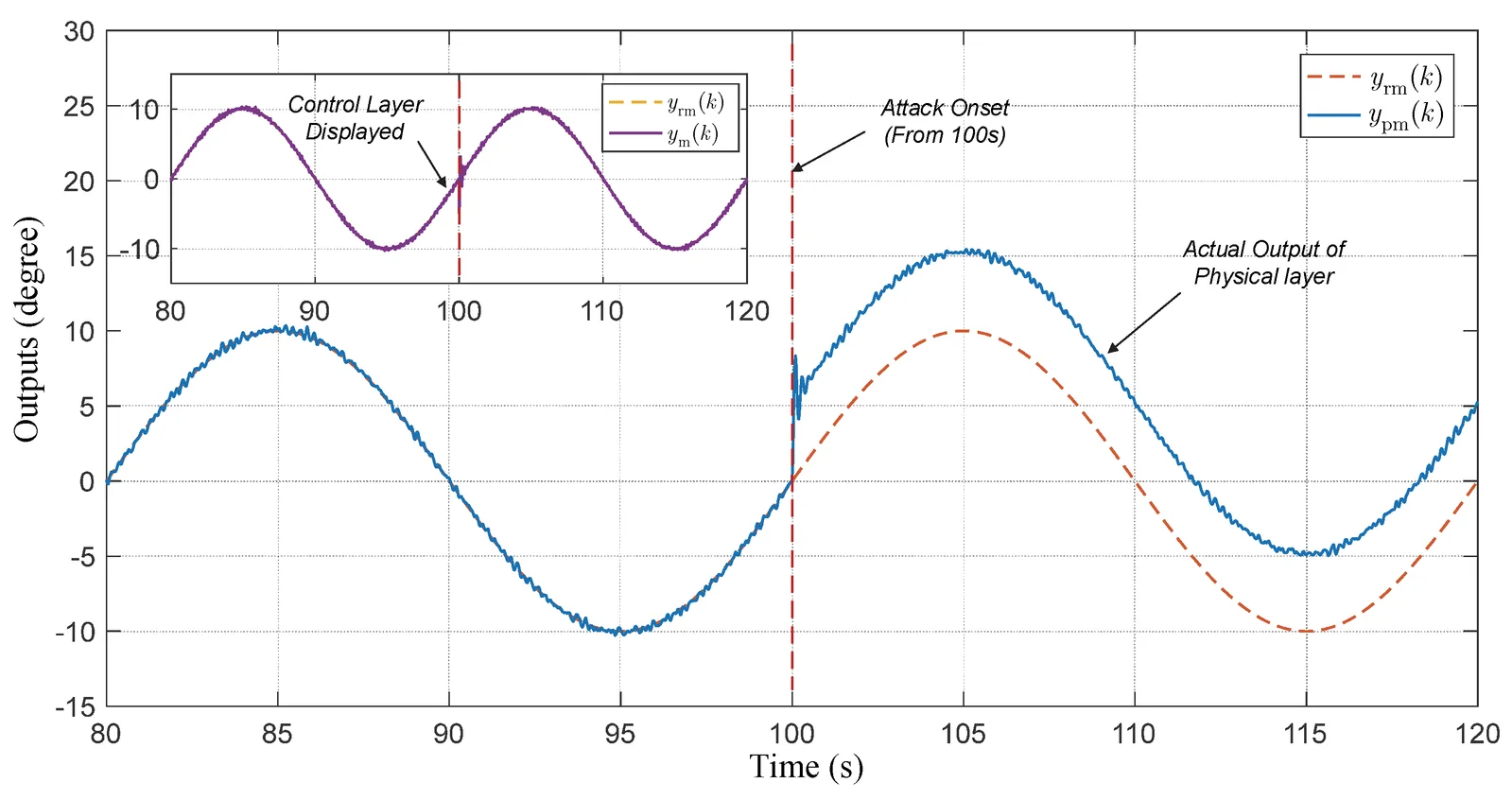
Output tracking control curve of the servo motor under FDI-I attacks.

**Figure 12:**
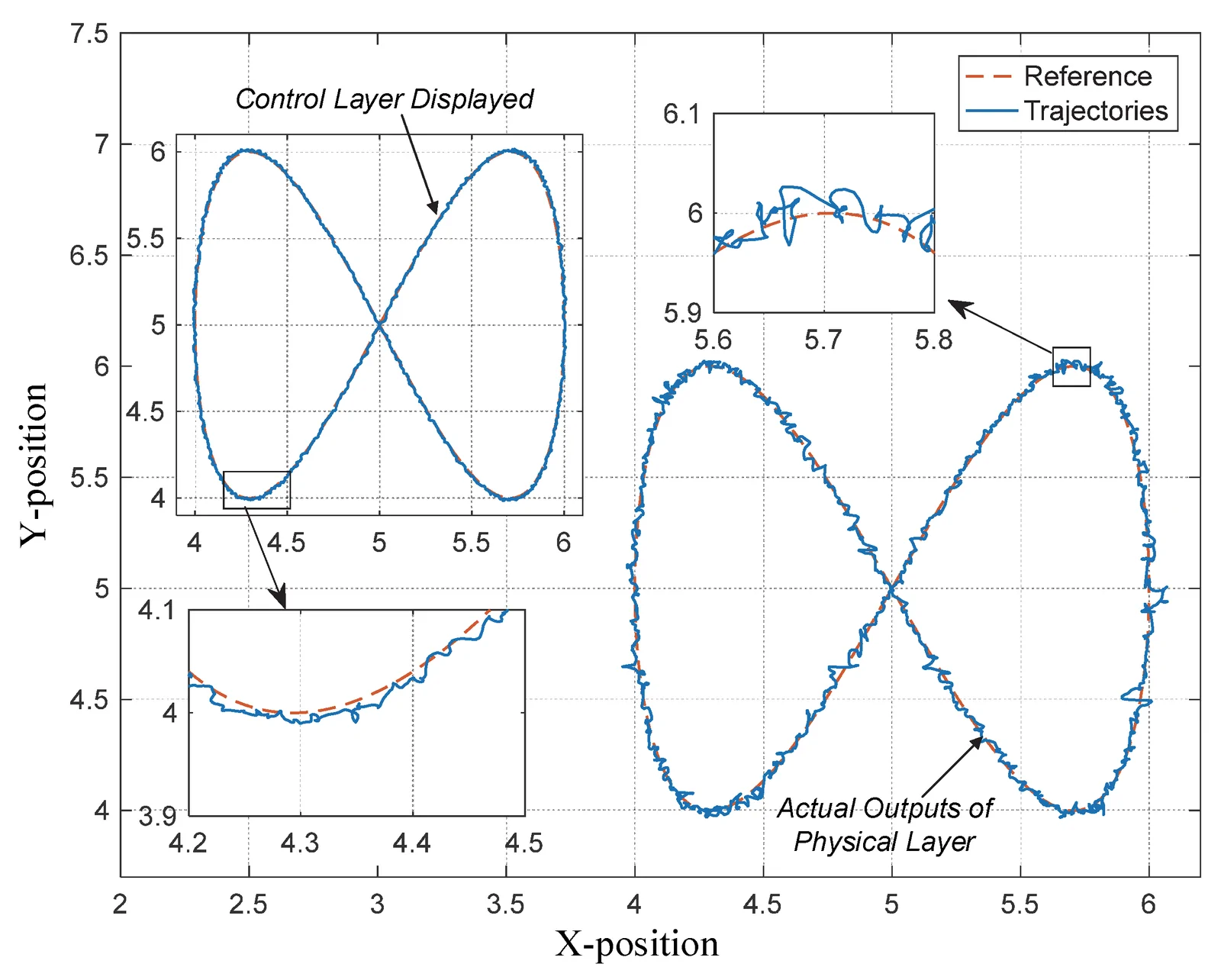
Trajectory tracking curves of the 2-DoF robot under FDI-II attacks.

Figure 11 illustrates the output tracking curves of the servo motor for 20 s before and after the attack. Under the FDI-I attack, the actual output of the physical system deviates from the predefined trajectory, yet no obvious anomaly appears in the physical layer output curve (displayed on the control layer), indicating that both the attack goal and stealthiness meet expectations. The trajectory tracking curve of the 2-DoF robot under FDI-II attack is given in Figure 12. Comparing the physical layer and control layer data clearly shows that this attack strategy also achieves the intended target while ensuring stealthiness.

**Figure 13:**
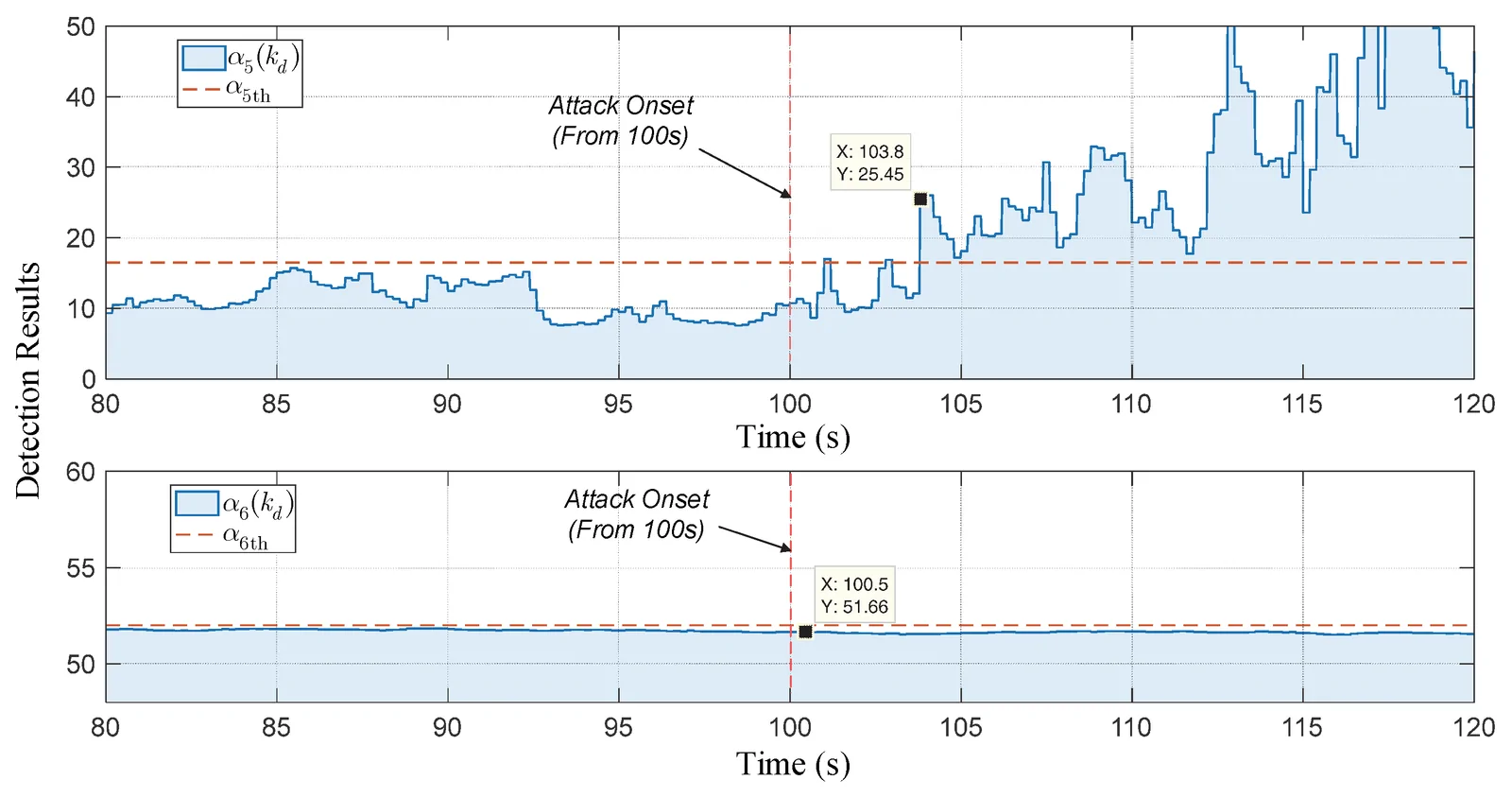
Detection results of the innovation detector for FDI-I and FDI-II attacks.

Regarding detection performance, Figure 13 shows the detection results of the innovation estimation detector against the two types of stealthy attacks. With the detection period set to 0.2 s, the detection variable $\alpha_{5}\left(k_{d}\right)$ exceeds its threshold $\alpha_{5th}=16.5$ starting from 103.8 s, demonstrating that the detector is effective against the FDI-I attack on the servo motor system. However, the detection results of the innovation estimation detector for FDI-II are shown by $\alpha_{6}\left(k_{d}\right)$ and $\alpha_{6th}=52$ in Figure 13. It can be observed that the detection variable shows no significant change before and after the attack, indicating that this detector fails to detect the FDI-II attack. The reason for this failure can be analyzed using the geometric interpretation in Section 3: on one hand, before and after the FDI-II attack, the subspace of the innovation sequence always maintains orthogonality with $M_{0}W_{f}$, ensuring that the estimation of $Z_{b}^{-}$ remains consistent (i.e., the variable $\alpha_{6}\left(k_{d}\right)$ always converges); on the other hand, the innovation term is numerically small, meaning that the base and hypotenuse of the triangle in the geometric space are nearly equal. Thus, the perturbation caused by FDI-II produces a minimal change in the orthogonal residual, ultimately rendering the detector blind to FDI-II attacks.

**Figure 14:**
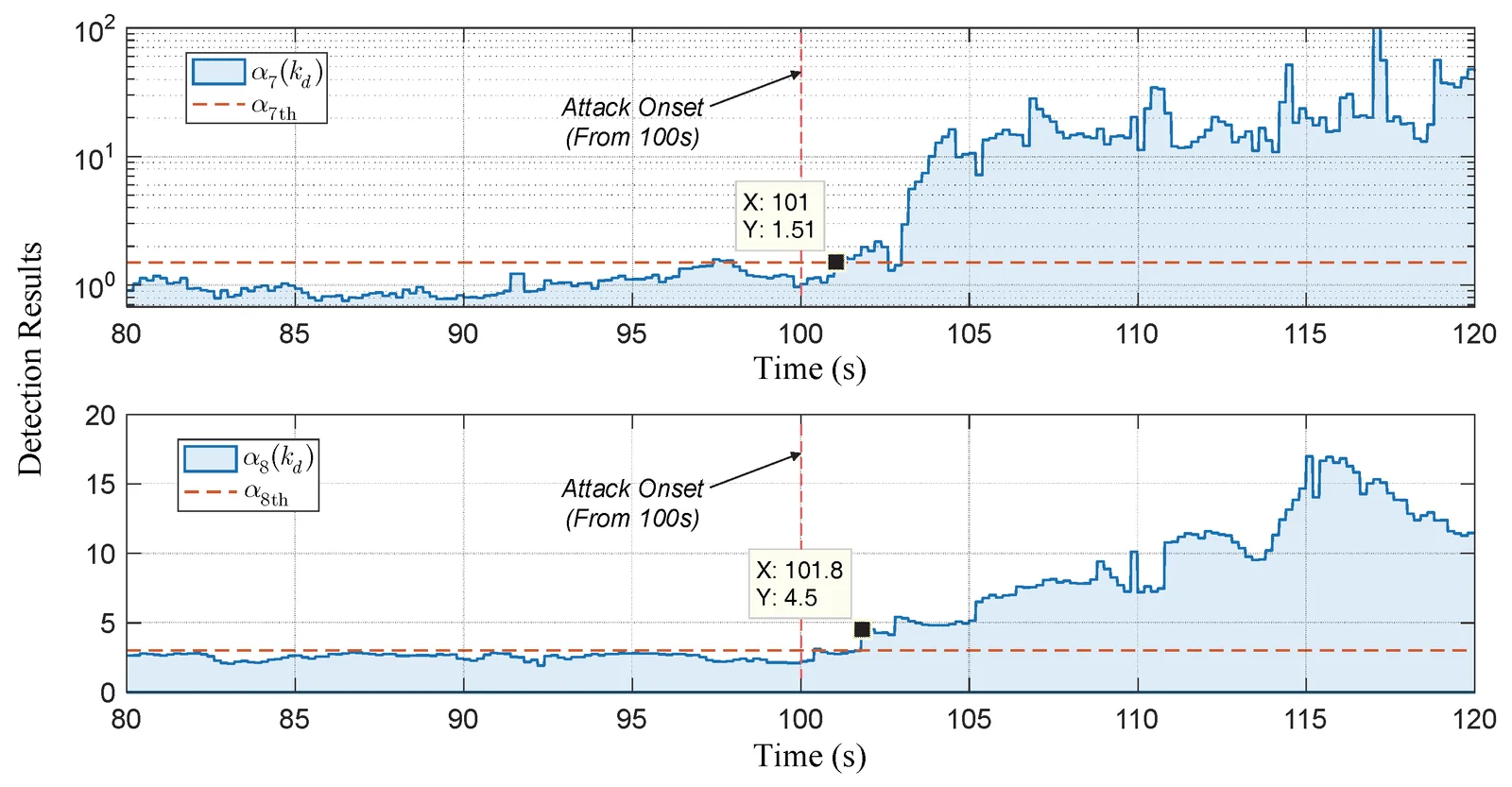
Detection results of the extended Markov detector for FDI-I and FDI-II attacks.

For the two types of stealthy FDI attacks, the detection performance of the extended Markov detector is presented in Figure 14. Evidently, the FDI-I and FDI-II attacks are detected at 101 s and 101.8 s, respectively, and maintain high detection variable outputs. These results indicate that the extended Markov detector is effective against both types of stealthy attacks. Comparing Figure 13 and Figure 14, it is clear that by introducing the dynamic quantification of the DGM, the extended Markov matrix reflects the causality of the process data, thereby maintaining detection capability even against attack strategies constructed using independent and identically distributed data.

## 5. Conclusions and Future Work

In this paper, a novel data-driven security detection framework based on DGM is proposed to safeguard ICPS against stealthy IA attacks. Building upon the multiplicative IA attack model presented in our previous work, this study relaxes the assumptions of known physical plant parameters and noise statistical properties that traditional residual-based detectors often rely on.

Using subspace matrix processing techniques, we construct two types of detection variables strictly from the operational data in the control layer. The static innovation sequence captures steady-state mismatches, showing high sensitivity against standard DoS and essential FDI attacks, as well as FDI-I attacks that can bypass conventional $\chi^{2}$ detectors. Furthermore, to address the more advanced FDI-II attacks that preserve the orthogonal relationship of the data subspace (and thus deceive KLD detectors), an extended Markov matrix estimator is proposed. Our geometric analysis explains that this dynamic detection variable effectively captures the rotational and stretching distortions of the subspace caused by malicious sequences. HIL experiments based on a 2-DoF robot and a DC servo motor verify the effectiveness of the proposed detection framework.

Future research will focus on extending the current linear DGM framework to non-linear and time-varying industrial processes. Additionally, while this study demonstrates the binary capability of breaking the undetectability of stealthy attacks via a custom HIL platform, evaluating precise quantitative statistical metrics including the false alarm rate, missed detection rate, and average detection delay remains challenging due to the current lack of universally standardized benchmark datasets in the ICPS domain. Therefore, establishing a comprehensive benchmarking methodology and investigating adaptive thresholds under severe exogenous disturbances, alongside exploring the distributed deployment of DGM detectors in large-scale networked CPS, represent promising directions to further improve industrial control security.

## Figures and Tables

**Table 1:** Nomenclature of main symbols used in Section 2.

Symbol	Description
$x_{p}\left(k\right),u_{p}\left(k\right),y_{p}\left(k\right)$	State vector, control input, and measurement output of the physical plant
$A_{p},B_{p},C_{p}$	Model parameter matrices of the physical plant
$w_{x}\left(k\right),w_{y}\left(k\right)$	Process noise and measurement noise
$n_{x},n_{u},n_{y}$	Dimensions of the state, input, and output variables
$x_{c}\left(k\right),u_{c}\left(k\right),y_{c}\left(k\right)$	State, output, and input variables of the tracking controller
$y_{r}\left(k\right),y_{e}\left(k\right)$	Desired output trajectory and output tracking error
$\Phi_{a}\left(k\right),\Phi_{s}\left(k\right)$	Time-varying attack matrices on the actuator and sensor channels

**Table 2:** Nomenclature of main symbols introduced in Section 3.

Symbol	Description
$\alpha \left(k\right),\alpha_{th}$	Real-time detection variable and the predefined detection threshold
*Q*	Positive definite weighting matrix
*s*	Length of the sliding data window
$U_{f},Y_{f},W_{f}$	Block-Hankel matrices of past inputs, past outputs, and combined past data
$U_{b},Y_{b}$	Block-Hankel matrices of recent inputs and recent outputs
$Z_{f},Z_{b},z\left(k\right)$	Block-Hankel matrices and the vector of the innovation sequence
p,q	Number of block rows and block columns in the Hankel matrices
Γ	Extended observability matrix
${\hat{C}}_{p}$	Estimated observation matrix under the subspace projection
$\hat{M}\left(k\right)$	Consistent estimate of the extended Markov matrix

## Data Availability

The original contributions presented in the study are included in the article; further inquiries can be directed to the corresponding author.

## Abbreviations

The following abbreviations are used in this manuscript:

2-DoF	Two Degree-of-Freedom
CPS	Cyber-Physical Systems
DC	Direct Current
DGM	Differences in Generalized Models
DoS	Denial-of-Service
FDI	False Data Injection
HIL	Hardware-in-the-Loop
IA	Integrity and Availability
ICPS	Industrial Cyber-Physical Systems
KLD	Kullback–Leibler Divergence
LTD	Long Time-series Data
LTI	Linear Time-Invariant
PID	Proportional Integral Derivative
UDP	User Datagram Protocol
WSCC	Western States Coordinated Council
